# Quebracho

**Published:** 1891-05-16

**Authors:** 


### THERAPEUTICS.

### QUEBRACHO.

Quebracho blanco, or the bark of the Aspidosperma quebracho, growing in the Argentine Republic, was introduced for medicinal purposes some years ago. The Quebracho is an Apocynaceous tree, growing about thirty-five or forty feet or more in height. The name, according to Dr. Rusby, is made up of two Spanish words—*quebra*, to break, and *hacha*, an axe. The name thus indicates a wood so hard as to endanger the axe used in cutting it. This fact, says Dr. Rusby, is not without its bearing on the confusion that has arisen from the mixing of specimens of quebracho wood, as it indicates how the name would be likely to become applied in different sections to different trees. In tropical America, trees producing a wood of this character are extremely common. Quoting Hieronymus, he tells us that the trunk and branches present the following appearance: The trunks of the oldest trees are clothed with a bark which has a greyish-brown surface with a reddish-brown ground, the inner bark of unchanged periderm being yellowish-white, but becoming yellowish, or often of a brick-red on drying in the air. The branches are originally of a greyish-green, but soon cover themselves with a grey or yellowish-brown, quite smooth, periderm. Hieronymus states that the natives of the Argentine Republic and Paraguay make many uses of the tree. The juice of the unripe fruit is said to possess rennet-like properties, and to be used in cheese making. Also that the bark and the leaves are used in tanning, but that the percentage of tannin varies considerably in different districts. Hieronymus further informs us that the wood, of white or yellow colour with light chocolate brown grain, is of great importance to the natives on account of its hardness and strength. The wood is used for tanning by the natives of South America, and it is said that in the fresh state a decoction of the wood is used internally for ague and malarial fever generally. Heise and Penzoldt, in their experimental investigations, found no alkaloids in the dried specimens, and showed that they possessed no therapeutical properties in malarial fevers. From the U.S. Dispensat. we find that Froude isolated aspidospermine from the bark of Quebracho blanco, and Hesse found five other alkaloids, aspidospermatine, and isomeric with it aspidosamine, and quebrachine, hypoquebrachine, and quebrachamine. Aspidospermine is soluble in about 6,000 parts of water, the solution having a very bitter taste, and it is soluble in about 48 parts of alcohol, in 106 parts of ether, and is insoluble in glycerine; it dissolves readily in fats and fixed oils, codliver oil dissolving a larger proportion than it does of quinine. Its sulphate and hydrochlorate are very soluble.

Of the six alkaloids we have mentioned as having been isolated from quebracho, quebrachine, aspidosamine, and aspidospermine are the most important, the first of these being the most active, the last is the least potent, and aspidosamine is intermediate in its degree of physiological potency. Harnack and Hofmann have found that in frogs paralysis of the respiratory centre rapidly follows the administration of these alkaloids. The central nervous system is paralysed, loss of voluntary movements from paralysis of the cortical motor centres being first observed, with an hyper excitability of the deep reflexes; finally the spinal cord is paralysed. All the alkaloids paralyse the voluntary muscles, and aspidosamine and hypoquebrachin also paralyse the motor nerve endings. The heart is at first slowed and finally paralysed, but in cold-blooded animals respiration is first affected. In warm-blooded animals cardiac paralysis occurs sooner than respiratory failure. Penzoldt concluded that quebracho possessed the property of favourably influencing respiratory affections characterized by insufficient oxidation, and he believed that this was due not only to its stimulating effect, but also to a power it possessed of increasing the affinity between hœmoglobin and oxygen. The experiments of many observers, in addition to those we have referred to above, do not bear out this view, and it is fairly established that it owes its therapeutical properties to its action on the nerve centres and motor nerve endings.

It is chiefly of value is spasmodic asthma, and the experiments of Schiffer have shown that of the various alkaloids, aspidospermine effects the respiration more markedly than any of the others. The alkaloid has been administered in this class of respiratory affections.

The dose of aspidospermine is from half to one grain. Aspidospermine in these small doses increases the amplitude of the respiratory movements. In larger doses, as stated above, respiratory paralysis results, preceded by irregularity of respiration. It thus appears to owe its beneficial action to its stimulating the respiratory centres, and may be compared to digitalis, which likewise acts on the vagus, the therapeutical properties of which are due to its stimulating the vagus, and which latter drug, if given in an overdose, paralyses the vagus centres. The tincture of quebracho may be given in doses of half to one fluid drachm. We have found it of great service in a patient who had used Himrod's powder for ten years almost every night, till it failed to produce relief.

Dr. Bourdeaux has found that the fluid extract of quebracho possesses physical and medicinal properties of great value in the treatment of superficial wounds. The extract, when painted on the wound, hardens in the course of an hour, forming a hard brown crust, which clings firmly to the tissues, and can only be removed by warm water. The exudations from the wound dry up, and healing rapidly takes place, after which the brown crust will fall off.

